# Characteristics of COVID-19 in cancer patients: a cross-sectional study in Peru

**DOI:** 10.3332/ecancer.2021.1246

**Published:** 2021-06-10

**Authors:** Eduardo Payet, Joan Perez, Gustavo Sarria, Silvia Neciosup, Francisco Berrospi, Sheila Vilchez, Jorge Dunstan, Ronald Perez, Mauricio Vassallo, Santiago Salgado, Nanto Caparachín, Joseph A Pinto, Alexis Holguin

**Affiliations:** 1 Departamento de Cirugía en Abdomen, Instituto Nacional de Enfermedades Neoplásicas, Av Angamos 2520, Surquillo, Lima 34, Peru; 2 Departamento de Cirugía Ginecológica, Instituto Nacional de Enfermedades Neoplásicas, Av Angamos 2520, Surquillo, Lima 34, Peru; 3 Departamento de Radioterapia, Instituto Nacional de Enfermedades Neoplásicas, Av Angamos 2520, Surquillo, Lima 34, Peru; 4 Departamento de Medicina Oncológica, Instituto Nacional de Enfermedades Neoplásicas, Av Angamos 2520, Surquillo, Lima 34, Peru; 5 Departamento de Cirugía en Mamas y Tejidos Blandos, Instituto Nacional de Enfermedades Neoplásicas, Av Angamos 2520, Surquillo, Lima 34, Peru; 6 Departamento de Medicina Crítica, Instituto Nacional de Enfermedades Neoplásicas, Av Angamos 2520, Surquillo, Lima 34, Peru; 7 Escuela Profesional de Medicina, Universidad Peruana de Ciencias Aplicadas, Av Prolongación Primavera 2390, Lima 15023, Peru; 8 Unidad de Gestión de Riesgos y Seguridad del Paciente, Instituto Nacional de Enfermedades Neoplásicas, Av Angamos 2520, Surquillo, Lima 34, Peru; 9 Escuela Profesional de Medicina Humana, Filial Ica, Universidad Privada San Juan Bautista, Carretera Panamericana Sur Km 300, Ica, 11004, Peru; 10 Departamento de Infectología, Instituto Nacional de Enfermedades Neoplásicas, Av Angamos 2520, Surquillo, Lima 34, Peru

**Keywords:** COVID-19, SARS-CoV-2, cancer, mortality

## Abstract

**Background:**

Cancer patients are at higher risk of infection and severity of Coronavirus Disease-19 (COVID-19). Management of patients infected by severe acute respiratory syndrome coronavirus 2 (SARS-CoV-2) is challenging due to the scarce scientific information and treatment guidelines. In this work, we present our Institutional experience with our first 100 patients with oncological malignancies and COVID-19.

**Patients and methods:**

We conducted a cross-sectional study of the first 100 patients hospitalised at the Instituto Nacional de Enfermedades Neoplasicas (Lima, Peru) who were positive for SARS-CoV-2 by reverse transcriptase (RT)-PCR during the period 30 March to 20 June. Clinicopathological variables of the oncological disease as well as risk factors, management and outcomes to COVID-19 were evaluated.

**Results:**

The mean age was 43.5 years old (standard deviations: ±24.8) where 57% were male patients. In total, 44%, 37% and 19% were adult patients bearing solid tumours, adults with haematologic malignancies and paediatric patients, respectively. Hypertension was the most frequent comorbidity (23%) followed by chronic lung disease (10%). COVID-19-associated symptoms included cough (65%), fever (57%) and dyspnoea (56%). Twelve percent of patients were asymptomatic. Nosocomial infections were more frequent in paediatric patients (84.2%) than in adult patients (16.0%). Patients with uncontrolled oncological disease were most frequent (72%). Anaemia was present in 67% of patients, 68% had lymphopenia, 62% had ferritin value > 500 mcg/L, 85% had elevated lactate dehydrogenase (LDH), 83% D-dimer > 500 ng/mL and 80% C-Reactive Protein > 8 mg/L. The most common complication was acute respiratory failure (42%). Overall fatality rate was 39% where the main cause of mortality was acute respiratory distress syndrome (64.1%).

**Conclusion:**

Paediatric patients had better outcomes than adult populations, and a high number of asymptomatic carriers and nosocomial infection, early diagnosis are recommended. Considering oncological treatments 30 days before COVID-19 diagnosis, our data did not reveal an increased mortality.

## Highlights

- A 39% mortality rate was observed where mortality in the group of adult patients was 50%.
- Acute Respiratory Distress Syndrome (ARDS) is the main cause of death in oncological patients with COVID-19.
- The major origin of SARS-CoV-2 infection in paediatric patients with cancer is nosocomial.

## Introduction

At the end of December 2019, 29 cases of pneumonia of unknown origin detected in Wuhan (China) were reported to the World Health Organization (WHO) [1, 2]. Further research identified the aetiological agent as a new coronavirus named later severe acute respiratory syndrome coronavirus 2 (SARS-CoV-2) and the causing disease as Coronavirus Disease-19 (COVID-19) [3, 4]. As the disease spreads across the globe, it was categorised as a pandemic on 11 March 2020 [3]. As of 24 October, more than 42 million of cases were diagnosed and 1.1 million of deaths have been reported worldwide (mortality rate of 2.7%) [5].

In Peru, the first case of COVID-19 was confirmed on 6 March 2020, in a Peruvian citizen who came from Europe. In the following months, SARS-CoV-2 spreads quickly in the country where some sociodemographic variables had a key role in the dissemination of this disease [6, 7]. As of October 2020, the Ministry of Health of Peru reported more than 883,000 cases (77.4% detected by rapid tests) and 34,000 deaths were reported (fatality rate of 3.85%). In total, 69.9% of deceased patients were older than 60 years old [8].

Cancer patients have an increased susceptibility and vulnerability to SARS-CoV-2 [9, 10]. A recent meta-analysis by Kong *et al* [11] showed that the incidence of severe illness in COVID-19 patients with cancer is 34% compared to 14% in cancer free individuals. On the other hand, the mortality rate was 20% compared to 0.05% in patients without cancer [11]. This increase in the fatality rate quickly positioned COVID-19 as a serious public health problem with life threatening complications for cancer patients [12]. In addition, cancer patients at Wuhan were 2.31 times more likely to contract the infection than non-cancer patients [13]. Cancer patients are twice more likely to have severe or critical symptoms, to need invasive mechanical ventilation (IMV) and consequently to be admitted to an intensive care unit (ICU) and eventually die [10].

In general, cancer patients have a worse prognosis and deteriorate quicker than a patient without cancer; moreover, SARS-CoV-2 infected cancer patients have a lower prevalence of IgG antibodies and they have a high risk to acquire a nosocomial infection of COVID-19 and a higher risk of death [10, 14, 15].

The COVID-19 pandemic has changed the global landscape of cancer management as well as for other chronic diseases where patients from developing countries and patients with scarce resources are the most affected. This current situation forces us to better understand the real impact of COVID-19 in our oncologic patients, as well as learn how COVID-19 behaves in cancer patients and how to improve the management of these patients and with the forthcoming economic crisis, our decision making in public health.

In this study, we aim to describe the features of COVID-19 in cancer patients who were hospitalised at our Institution as well as the treatment strategies and outcomes in these patients.

## Patients and methods

### Study design and patient’s population

We conducted a retrospective, descriptive and cross-sectional study to describe the clinicopathological characteristics, risk factors, treatments and outcomes of cancer patients diagnosed with COVID-19 infection. We included paediatric and adult cancer patients hospitalised (at least 24 hours) at the Instituto Nacional de Enfermedades Neoplásicas (INEN) during the period 30 March–20 June 2020 and positive results for RNA detection of SARS-CoV-2 by RT-PCR.

### Variables and outcomes

We evaluated baseline cancer-related variables including age (at COVID-19 diagnosis), sex, comorbidities, smoking status, type of cancer, clinical stage, control of oncological disease (controlled disease was defined as absence of cancer progression or recurrence after treatment), previous treatment for cancer (prior 30 days). COVID-19-related variables included initial signs and symptoms, severity of COVID-19 (as described initially by the WHO [16]), origin of infection (according to qualified by Wang *et al* [17]), main findings on computed tomography (CT) study, percentage of lung involvement as evaluated in the CT study (according to Bernheim *et al* [18]) and results of bronchioalveolar lavage (BAL), blood and urine culture. Variables related to the management of COVID-19 disease included the maximum oxygen requirement (We define low oxygen requirement as the use of binasal cannula and simple mask, and high oxygen requirement as the use of reservoir mask), admission to the ICU, pharmacological treatment for COVID-19, antibiotic treatment to companion infections, drug-related toxicities. Finally, COVID-19 assessed outcomes included complications, clinical outcome, fatality rate and cause of death.

### Statistical analysis

Due to the small sample size, we conducted only descriptive studies. For quantitative variables, means and standard deviations (SD) were estimated. For qualitative variables, we calculated frequencies and percentages.

### Ethical considerations

This study was reviewed and approved by the Institutional Review Board (IRB) of the INEN (approval 049-2020-CRPI-DI-DICON/INEN). The use of an informed consent was waived by the IRB.

## Results

### Patient demographic and tumour characteristics

During the study period, 100 patients met our eligibility criteria where 44% of patients were adults with solid tumours, 37% were adults with haematological malignancies and 19% were paediatric cancers. The mean age was 43.5 years old (SD: ±24.8; range: 18 months to 80 years old); in paediatric patients, the mean age was 5 years old (SD: ±4.4), while in adults it was 52.4 years old (SD: ±17.6). Fifty-seven percent of patients were male. Regarding to comorbidities, 23% of patients had hypertension, 10% chronic lung disease, 8% obesity and 6% diabetes. Eight percent of patients were smokers. The most frequent type of cancers was leukaemia (28%), lymphomas (19%), breast and soft tumours (12%), among others. In total, 40% of patients received chemotherapy, 7% surgery and 4% radiotherapy within 30 days prior to COVID-19 diagnosis. A detailed description of the patient’s characteristics is presented in Table 1.

### Clinical presentation of COVID-19

Cough, fever, dyspnoea and tachypnoea were the most frequent initial signs and symptoms (65%, 57%, 56% and 56%, respectively). Regarding WHO COVID-19 interim guidance, severity was assessed as 32% were mild; 33%, moderate; 17%, severe and 18% were critical patients [16]. Overall, 71% of infections were in the community and 29% were nosocomial infections. Interestingly, 84.2% of COVID-19 infections in paediatric patients were acquired during the hospitalisation. In patients with chest-CT evaluation, main findings were grounded-glass opacities (55%) and consolidative pulmonary opacities (32%). Twenty-four percent of cases had a lung involvement > 51%. A more detailed description of the COVID-19 presentation is presented in Table 2.

**Figure 1.**
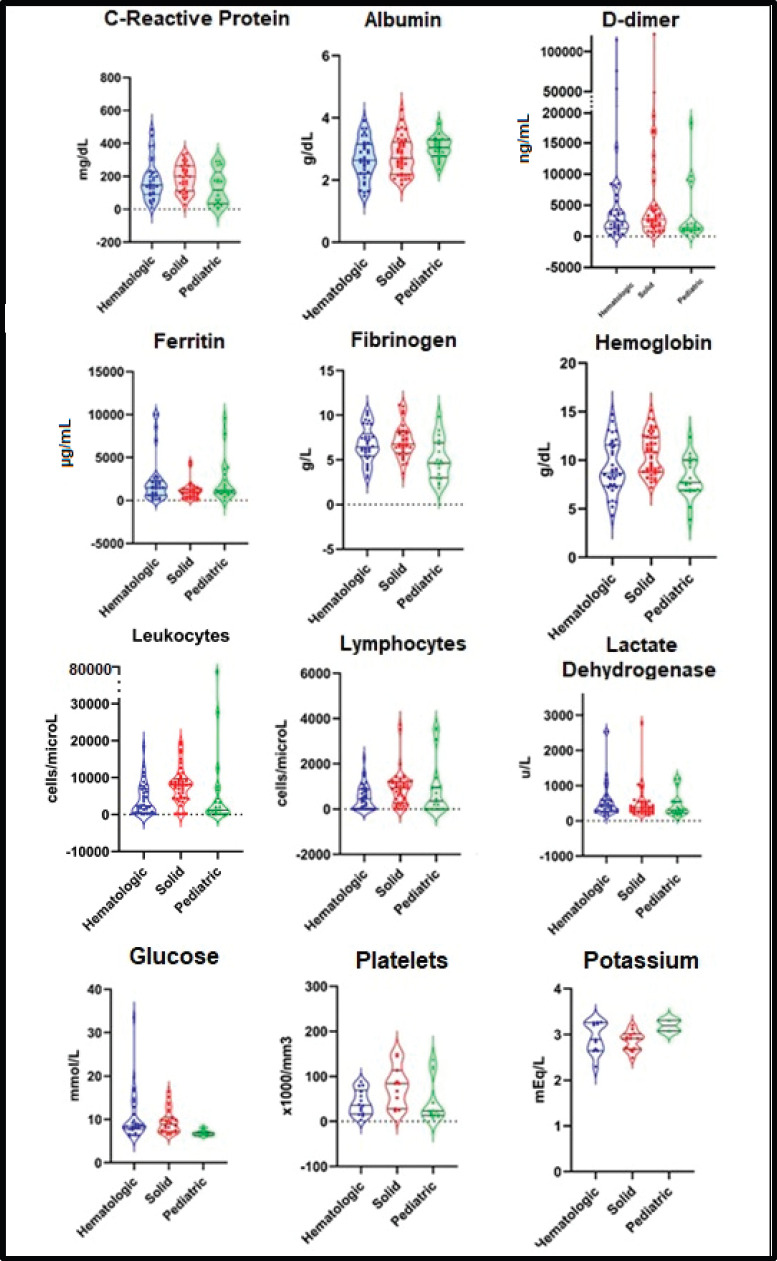
Main findings in laboratory parameters in patients with COVID-19 and cancer.

The mean concentration of the main laboratory parameters was C-reactive, 173.6 mg/L (SD: ±104.7 mg/L)M; D-dimer, 8,177.8 ng/mL (SD: ±19,225.0 ng/mL); ferritin, 1,821.1 μg/mL (±2,243.4 μg/mL); fibrinogen, 6.5 g/L (±2.1 g/L) and lactate dehydrogenase 525.0 U/L (±468.2 U/L). A detailed description of biochemical findings is presented in Figure 1.

### Treatment for COVID-19 and complications

In total, 11% of patients required IMV while 31% required high flow of O_2_. Twelve percent of patients had admission to ICU (11 adults and 1 paediatric patient). The most frequent drugs indicated to COVID-19 management were enoxaparin (49%), methylprednisolone (37%), dexamethasone (25%), azithromycin (12%), ivermectin (12%) and hydroxychloroquine (9%). The antibiotic treatment to co-infections included piperacillin/tazobactam (60%), meropenem (43%) among others. Drug-related toxicities included hyperglycaemia in 54% of patients, hypokalaemia in 4% and hyponatraemia in 1% of cases (Table 3).

### Clinical course and outcomes

Regarding the clinical course of COVID-19, 42% of patients developed acute respiratory failure (50.6% in adults versus 1% in paediatrics), 19% had ARDS (23.5% in adults versus 0% in paediatrics), 12% had septic shock and other complications (14.8% in adults versus 0% in paediatrics). Forty-eight percent of patients (43% in adults versus 68% in paediatrics) had been discharged at the end of follow-up period (mean of hospital stay in adults, 15.9 days, SD: ±10.9 versus 11.9 days, SD: ±10.5, in paediatric patients). Overall, the fatality rate was 39%, 50% in adult patients with solid tumours, 43.2% in adults with haematologic tumours and 5.3% in paediatric patients. The cause of death was ARDS in 64.1%, septic shock in 20.5%, multi-organ failure in 10.3%, cardiorespiratory arrest in 2.6% and progression of oncological disease in 2.6% (Table 4).

## Discussion

The ongoing COVID-19 pandemic has had terrible consequences to the Peruvian public health [19]. This public emergency caused the delay of treatment of patients with chronic diseases, including cancer.

Since the beginning of the pandemic, INEN established multiple actions to guarantee adequate protection for patients and healthcare personnel, while ensuring continuity of cancer care. Among these measures, we fully endorsed the recommendations of the Ministry of Health of Peru and the WHO. In order to detect early suspicious cases of COVID-19, a differentiated triage was established to identify persons under investigation and help guide initial screening tests [20, 21]. In this work, we present our experience managing cancer patients at INEN, the largest hospital for specialised cancer care in Peru. Because of the limited number of patients and events (39 deaths), we did not conduct association studies. On the other hand, the characteristics of our patients could, including inclusion of paediatric patients, lead to some differences with previous reports.

The mean age at diagnosis of COVID-19 in our patients was younger (43.5 years old; SD: ±24.8) than the mean age reported in other similar studies, although we included paediatric patients [3, 22, 23]. Comorbidities were present in 57% of our patients where 23% of them had hypertension. Previous report have described similar rate of comorbidities between COVID-19 patients with cancer and without cancer [10].

Haematological malignancies were the most frequent types of cancer (leukaemia and lymphoma), despite according to our epidemiology, solid tumours are most prevalent [24]. This contrast with two previous reports from China where they described lung malignancies as the most prevalent cancer among COVID-19 patients (20%–25%) [3, 10]. It could be explained by the high lung cancer incidence in China [24]. In addition, comparing medical consultations between March and June of 2019 versus 2020, the department of epidemiology and statistics of INEN report a decrease of 51.6% and 79.6% in haematological and solid cancer attentions, respectively.

Cough was the most common symptom at the COVID-19 setting in our patients. This differs from the reviewed studies, in which fever was seen as the most common symptom on admission both in patients with (94%) or without (82.1%) cancer [25]. It could be explained by the considerable percentage of asymptomatic patients that was described in our study (12%).

Overall, 29% of patients had nosocomial SARS-CoV-2 infection. Interestingly, while ≈80% of adults acquired the COVID-19 in the community, more than 80% of paediatric patients were infected in the hospital. Our Institutional policy for hospitalised paediatric patients is the joint accommodations of the patients with their guardian. The parents could have *ad libitum* access. Nosocomial transmission of COVID-19 among non-cancer patients only represents 1.49% of infections [10]. Previous reports in cancer patients show nosocomial infection rates of 19%–28.6% [3, 10].

Our rate of patients admitted to ICU was low (10.48%). As Institutional protocol, initiation of best supportive care and less invasive care measures were encouraged for patients with baseline uncontrolled disease or advanced clinical stages, which was based on utilitarian principlism due to scarcity of ICU beds. This de-escalation approach could explain the difference in the ICU admission rate compared to other international publications (7%–21.4%) [3, 10, 26].

Management guidelines for COVID-19 have been continuously changing during the pandemic. Until 4 of July, the WHO no longer recommends the use of hydroxychloroquine [16, 27]; before this recommendation, it was administered to 9% of our patients as prophylaxis. The use of enoxaparin is recommended as prophylaxis for deep vein thrombosis and was given to a half of our patients [16, 27]. Systemic anticoagulation was not administered to our paediatric patients as the WHO does not recommend it [16, 27]. The RECOVERY trial showed that the use of systemic corticosteroids reduces mortality up to a third of patients with respiratory complications during the hyperinflammatory phase [27, 28]. In our cohort, dexamethasone was used in 25% of patients, methylprednisolone in 37% and other corticosteroids in 16% of cases, including prednisone and hydrocortisone. On 16 June 2020, the University of Oxford announced the positive effects of the use of dexamethasone, being close to the end of data collection [28].

More than half of our patients (52%) developed complications, highlighting acute respiratory failure (42%), ARDS (19%) and septic shock (12%). On the other hand, we had a higher mortality rate in comparison to other similar studies (11.43%–28.6%) [3, 10, 26]. It should be noted that in our cohort, we had a 72% of patients with uncontrolled oncological disease; in addition, mortality in patients admitted to ICU was 75%.

Patients who have received prior cancer treatment are more susceptible to infection than patients without cancer. Patients treated with surgery and chemotherapy have five times higher risk of a severe clinical event than those who did not receive any treatment [29]. In our study, 30.9% of the patients who received cancer treatment died. This could differ from previously mentioned study, the mortality rate found in patients that received chemotherapy (25%) or had surgery (28.6%) was lower than the fatality rate. On the other hand, the mortality rate of patients that did not receive cancer treatment in the last 30 days was 48.5%. Prospective and well-powered studies and meta-analysis of observational studies have not related the type of treatment with mortality by COVID-19 in cancer patients [26, 30].

## Conclusion

In conclusion, paediatric patients had a lower mortality rate than adult populations and a higher number of asymptomatic carriers, early diagnosis and continuous testing in this group are recommended. In addition, strict protocols are encouraged to avoid intrahospitalary infections. Finally, considering oncological treatments 30 days before COVID-19 diagnosis, our data did not reveal an increased mortality, similar to worldwide publications. Conversely, they had lower mortality rates than the rest of the patients.

## Conflicts of interest

The authors declare that they have no conflict of interests with this research.

## Funding

None.

## Figures and Tables

**Figure 2.**
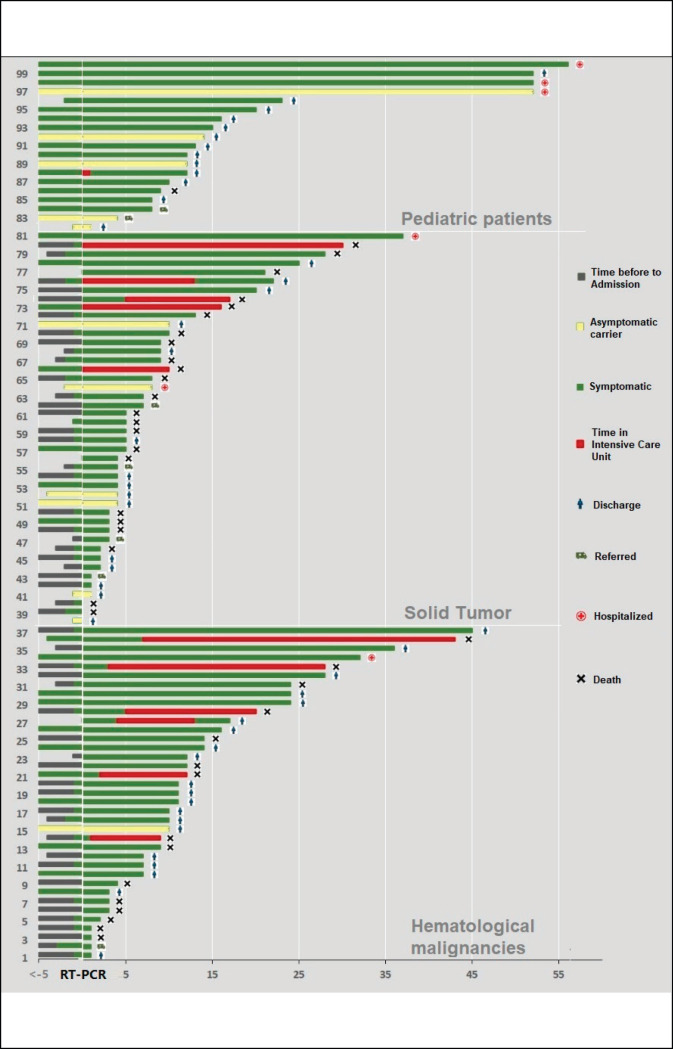
Timeline of the evolution of patient for type of cancer.

**Table 1.** Patient demographics and tumour characteristics.

Characteristics	All patients (*N* = 100)	Haematological malignancies (*n* = 37)	Solid tumours (*n* = 44)	Paediatric patients (*n* = 19)
	*N* (%)	*n* (%)	*n* (%)	*n* (%)
**Age** Mean (±SD)	43.5 (24.8)	49 (17.9)	55.3 (18)	5.2 (4.3)
**Age groups** <15 15–20 21–30 31–40 41–50 51–60 61–70 >70	19 (19) 8 (8) 5 (5) 6 (6) 16 (16) 10 (10) 22 (22) 14 (14)	0 5 (13.5) 1 (2.7) 5 (13.5) 8 (21.6) 6 (16.2) 8 (21.6) 4 (10.8)	0 3 (6.8) 4 (9.1) 1 (2.3) 8 (18.2) 4 (9.1) 14 (31.8) 10 (22.7)	19 (100.0) 0 0 0 0 0 0 0
**Sex** Male Female	57 (57) 43 (43)	21 (56.8) 16 (43.2)	23 (52.3) 21 (47.7)	13 (68.4) 6 (31.6)
**Comorbidities^a^** None Hypertension Chronic lung disease Obesity Diabetes mellitus Chronic liver disease Others	43 (43) 23 (23) 10 (10) 8 (8) 6 (6) 1 (1) 34 (34)	13 (35.1) 8 (21.6) 6 (16.2) 5 (13.5) 3 (8.1) 0 15 (40.5)	21 (47.7) 13 (29.5) 3 (6.8) 3 (6.8) 3 (6.8) 1 (2.3) 11 (25.0)	9 (47.4) 2 (10.5) 1 (5.3) 0 0 0 8 (42.1)
**Smoking status** No yes	92 (92) 8 (8)	33 (89.2) 4 (10.8)	40 (90.9) 4 (9.1)	19 (100) 0
**Type of cancer** Lymphoma Multiple myeloma Leukaemia Breasts and soft tumours Urological Gastrointestinal Central nervous system Head and neck Lung Unknown primary metastasis Gynaecologic Osteosarcoma Edwing’s sarcoma Hepatoblastoma Wilms tumour Rhabdomyosarcoma	19 (19) 3 (3) 28 (28) 12 (12) 12 (12) 8 (8) 4 (4) 3 (3) 2 (2) 1 (1) 1 (1) 2 (2) 2 (2) 1 (1) 1 (1) 1 (1)	19 (51.4) 3 (8.1) 15 (40.5) 0 0 0 0 0 0 0 0 0 0 0 0 0	0 0 0 12 (27.3) 12 (27.3) 8 (18.2) 3 (6.8) 3 (6.8) 2 (4.5) 1 (2.3) 1 (2.3) 1 (2.3) 1(2.3) 0 0 0	0 0 13 (65.0) 0 0 0 1 (5.3) 0 0 0 0 1 (5.3) 1 (5.3) 1 (5.3) 1 (5.3) 1 (5.3)
**Controlled oncological disease** No Yes	72 (72) 28 (28)	28(75.7) 9 (24.3)	28 (63.6) 16 (36.4)	16 (84.2) 3 (15.8)
**Oncologic treatment in the last 30 days^a^** No Surgery Chemotherapy Radiotherapy Hormonotherapy	45 (45) 7 (7) 40 (40) 4 (4) 6 (6)	17 (45.9) 1 (2.7) 18 (48.6) 1 (2.7) 0	23 (52.3) 5 (11.4) 8 (18.2) 3 (6.8) 6 (13.6)	5 (26.3) 1 (5.3) 14 (73.7) 0 0

a Variables with one or more answers

**Table 2.** Clinical presentation of COVID-19.

Initial clinical features	All patients (*N* = 100)	Haematological malignancies (*n* = 37)	Solid tumours (*n* = 44)	Paediatric patients (*n* = 19)
	*N* (%)	*n* (%)	*n* (%)	*n* (%)
**Initial signs and symptoms** Cough Fever Dyspnoea Tachypnoea Fatigue Diarrhoea Myalgia Chest pain Anosmia Asymptomatic	65 (65) 57 (57) 56 (56) 56 (56) 41 (41) 12 (12) 9 (9) 7 (7) 2 (2) 12 (12)	30 (81.1) 23 (62.2) 28 (75.7) 22 (59.5) 26 (70.3) 9 (24.3) 4 (10.8) 3 (8.1) 2 (5.4) 1 (2.7)	30 (68.2) 24 (54.5) 26 (59.1) 27 (61.4) 14 (31.8) 3 (6.8) 4 (9.1) 4 (9.1) 0 6 (13.6)	5 (26.3) 10 (52.6) 2 (10.5) 7 (36.8) 1 (5.3) 0 1 (5.3) 0 0 5 (26.3)
**Severity** Mild Moderate Severe Critical	32 (32) 33 (33) 17 (17) 18 (18)	8 (21.6) 16 (43.2) 5 (13.5) 8 (21.6)	13 (29.5) 10 (22.7) 12 (27.3) 9 (20.5)	11 (57.9) 7 (36.8) 0 1 (5.3)
**Origin of infection** Community Nosocomial	71 (71) 29 (29)	30 (81.1) 7 (18.9)	38 (86.4) 6 (13.6)	3 (15.8) 16 (84.2)
**CT study** No Yes	11 (11) 89 (89)	1 (2.7) 36 (97.3)	3 (6.8) 41 (93.2)	7 (36.8) 12 (63.2)
**Main finding on CT imaging** Normal Consolidative Pleural effusion Nodular Ground glass	7 (7) 29 (32) 1 (1) 3 (3) 49 (55)	4 (11.1) 13 (36.1) 0 1 (2.8) 18 (50.0)	0 13 (31.7) 1 (2.4) 0 27 (65.9)	3 (25) 3 (25) 0 2 (16.7) 4 (33.3)
**Nosocomial infections**				
**Blood culture^a^** Coagulase (−) Staphylococcus Enterobacteria *Acinetobacter baumannii* *C. albicans* Not applicable	5 (5) 1 (1) 1 (1) 1 (1) 92 (92)	3 (8.1) 1 (2.7) 0 0 33 (89.2)	1 (2.3) 0 0 1 (2.3) 42 (95.5)	1 (5.3) 0 1 (5.3) 0 17 (89.5)
**BAL culture^a^** Klebsiella *S. maltophilia* Not done	3 (3) 1 (1) 96 (96)	0 1 (2.7) 36 (97.3)	3 (6.8) 0 41 (93.2)	0 0 19 (100.0)
**Urine culture^a^** *C. tropicalis* Enterococcus Pseudomonas Not done	1 (1) 1 (1) 2 (2) 96 (96)	1 (2.7) 1 (2.7) 0 35 (94.6)	0 0 1 (2.3) 42 (97.7)	0 0 1 (5.3) 18 (94.7)

a Variables with one or more answers

**Table 3.** Management of COVID-19 in cancer patients.

Characteristics	All patients (*N* = 100)	Haematological malignancies (*n* = 37)	Solid tumours (*n* = 44)	Paediatric patients (*n* = 19)
	*N* (%)	*N* (%)	*N* (%)	*N* (%)
**Maximum oxygen requirement** Not require Low flow High flow IMV	39 (39) 19 (19) 31 (31) 11 (11)	8 (21.6) 10 (27.0) 13 (35.1) 6 (16.2)	14 (31.8) 9 (20.5) 16 (36.4) 5 (11.4)	17 (89.5) 0 2 (10.5) 0
**Admission to ICU** No Yes	88 (88) 12 (12)	31 (83.8) 6 (16.2)	39 (88.6) 5 (11.4)	18 (94.7) 1 (5.3)
**Pharmacological treatment for COVID-19^a^** Enoxaparin Hydroxychloroquine Azithromycin Ivermectin Dexamethasone Methylprednisolone Others	49 (49) 9 (9) 12 (12) 10 (10) 25 (25) 37 (37) 16 (16)	24 (64.9) 6 (16.2) 4 (10.8) 6 (16.2) 11 (29.7) 16 (43.2) 7 (18.9)	25 (56.8) 3 (6.8) 5 (11.4) 3 (6.8) 11 (25.0) 18 (40.9) 7 (15.9)	0 0 3 (15.8) 1 (5.3) 3 (15.8) 3 (15.8) 2 (10.5)
**Antibiotic treatment^a^** Piperacillin/Tazobactam Vancomycin Meropenem Others	60 (60) 40 (40) 43 (43) 23 (23)	30 (81.1) 17 (45.9) 17 (45.9) 7 (18.9)	26 (59.1) 16 (36.4) 17 (38.6) 11 (25.0)	4 (21.1) 7 (36.8) 9 (47.4) 2 (10.5)
**Drug-related toxicities^a^** None Hyperglycaemia Hyponatraemia Hypokalaemia	44 (44) 54 (54) 1 (1) 4 (4)	10 (27) 27 (73) 1 (2.7) 4 (10.8)	20 (45.5) 24 (54.5) 0 0	14 (73.7) 5 (26.3) 0 0

a Variables with one or more answers

**Table 4.** Clinical outcomes of patients diagnosed with a neoplasm and COVID-19.

Characteristics	All patients (*N* = 100)	Haematological malignancies (*n* = 37)	Solid tumours (*n* = 44)	Paediatric patients (*n* = 19)
	*N* (%)	*n* (%)	*n* (%)	*n* (%)
**Complications^a^** Acute respiratory failure ARDS Septic shock Sepsis Distributive shock Acute renal failure Multifactorial encephalopathy Tracheostomy None	42 (42) 19 (19) 12 (12) 8 (8) 4 (4) 2 (2) 2 (1) 1 (1) 48 (48)	20 (54.1) 11 (29.7) 5 (13.5) 2 (5.4) 3 (8.1) 2 (5.4) 1 (2.7) 0 13 (35.1)	21 (47.7) 8 (18.2) 7 (15.9) 6 (13.6) 1 (2.3) 0 1 (2.3) 1 (2.3) 17 (38.6)	1 (5.3) 0 0 0 0 0 0 0 18 (94.7)
**Clinical outcome** Discharge Death Referred Remaining hospitalised	48 (48) 39 (39) 7 (7) 6 (6)	19 (51.4) 16 (43.2) 1 (2.7) 1 (2.7)	16 (36.4) 22 (50.0) 4 (9.1) 2 (4.5)	13 (68.4) 1 (5.3) 2 (10.5) 3 (15.8)
**Days to discharge** Mean (±SD)	13(10.9)	15.8 (10.7)	7.2 (7.6)	15.9 (11.6)
**Fatality rate**	39%	43.2%	50.0%	5.3%
**Cause of death** ARDS Septic shock Multi-organ failure Cardiorespiratory arrest Progression of oncological disease	25 (64.1) 8 (20.5) 4 (10.3) 1 (2.6) 1 (2.6)	12 (75) 3 (18.8) 0 1 (6.3) 0	12 (54.5) 5 (22.7) 4 (18.2) 1 (5) 1 (4.5)	1 (100) 0 0 0 0

a Variables with one or more answers
